# Synchronous Online Journal Club to Connect Subspecialty Trainees across Geographic Barriers

**DOI:** 10.5811/westjem.2019.7.43545

**Published:** 2020-12-09

**Authors:** Andrew N. Musits, Alexandra L. Mannix

**Affiliations:** * Brown University Warren Alpert Medical School, Department of Emergency Medicine, Providence, Rhode Island; † University of Florida College of Medicine-Jacksonville, Department of Emergency Medicine, Jacksonville, Florida

## Abstract

**Introduction:**

Journal club holds a well-respected place in medical education by promoting critical review of the literature and fostering scholarly discussions. Journal clubs are often not available to trainees with niche interests due to the geographic limitations of subspecialty programs such as simulation, medical education, disaster medicine, ultrasound, global health, and women’s health.

**Methods:**

A recurring online journal club was held on a quarterly basis to connect simulation fellows. An online conferencing program with screen-sharing capabilities served as the platform for this scholarly exchange. Articles were presented by fellows supported by more seasoned mentors. We surveyed participants to evaluate the program and provide feedback to the presenter.

**Results:**

The first eight sessions drew participants from across the United States and Canada. The program was highly rated by participants who commented specifically on its value. Presenters were also highly rated, suggesting that fellows, with online support and mentoring, were effective in providing a quality program.

**Conclusion:**

Online synchronous journal clubs can fill an educational niche for subspecialists and their trainees, as demonstrated with this curriculum piloted with simulation fellows. Challenges of scheduling across time zones, distribution of materials, and recruitment of participants can be overcome by a dedicated team of facilitators aided by readily accessible technology.

## INTRODUCTION

The practice of journal clubs at academic medical centers began over 100 years ago. Since then, journal clubs have grown to hold a well-respected role in continuing medical education.^1^ Journal clubs are now common across many fields of medicine benefiting everyone from physicians in training to participants from pharmacy and nursing.^2^ The practice is popular across subspecialties in medicine and occurs throughout the world. Journal clubs have been shown to be effective in improving knowledge and critical appraisal skills.^3^

After residency training, some trainees will continue to build a niche through fellowship training. The educational innovation described in this report was piloted with simulation fellows. Simulation fellows dedicate their fellowship training to learning the theory behind and practical strategies to implement successful simulation-based medical education and research. Due to the niche of simulation training, fellows are often isolated from like-minded scholars. Local interaction with others in simulation may be limited due to low numbers of faculty trained in simulation, and few passionate simulation experts at their institution. A baseline needs assessment from participants demonstrated that 73% of the participants had, on average, two or less communications per month with simulation colleagues at other centers, and 55% reported they did not have access to a local simulation journal club.

One might extrapolate that a similar need exists in other subspecialties such as medical education, disaster medicine, ultrasound, women’s health, and global health, to name a few. Furthermore, medical students and other trainees may attend institutions where local experts do not exist. For example, a medical student may have an interest in global health but attend a school that does not have a global health program or faculty with this expertise. A synchronous online journal club, as described below, can provide exposure and networking opportunities not locally available. Program goals include exposing simulation fellows to advances in simulation-based education and research outside of their locality and clinical specialty, improving knowledge and critical appraisal of current research, and increasing communication and collaboration among simulation fellows and professionals at different geographic sites.

## OBJECTIVES

Through regular participation in this activity, learners will be able to do the following: 1) Identify advances and new trends in their field, occurring outside of their locality; 2) demonstrate critical appraisal of simulation literature; and 3) exhibit increased scholarly communication with colleagues at distant sites.

## CURRICULAR DESIGN

In-person journal clubs typically begin with a brief presentation of the article followed by an analytical discussion of methods and how to interpret the results and conclusions. This same structure was followed for the synchronous online journal club. A different simulation fellow presented at each session. He or she chose the article and prepared PowerPoint slides to visually aid the presentation. Presenters connected with a more experienced mentor to help screen appropriate articles and serve as a resource for the trainee preparing the presentation. The article chosen by the presenter was announced to the participants by e-mail 1–2 weeks prior to the live session. The full citation and a link to the article were often included. Unless the article was open access or freely available, the PDF was not included. The other participants in the session were other fellows and scholarly-minded simulation educators/researchers not in a fellowship role.

Presenters were provided with a slide template and encouraged to present using a standardized format. The first 20 minutes consisted of a factual presentation of relevant background, methods, results, and the author’s conclusions. The next 30 minutes was used for discussion. Presenters were encouraged to have specific questions and discussion topics to help facilitate and guide the discussion. In lieu of a traditional in-person meeting, the conversation was through an online conferencing program, thus eliminating geographic barriers. This recurring program met quarterly.

**Population Health Research Capsule**

What do we already know about this issue?

*Journal club is a common educational activity that fosters critical literature appraisal. Subspecialty fellows may not have local access to this opportunity*.

What was the research question?

Can an online journal club connect geographically distant trainees for discussion of subspecialty literature?

What was the major finding of the study?

*An eight-session pilot with simulation fellows had 83 learner encounters. It was feasible and well received*.

How does this improve population health?

*Online journal club may be applied to other subspecialties to complement education, provide presentation opportunities, and increase networking*.

An online conferencing system was used to facilitate the online discussion and allow the presenter to share his or her screen. Several similar platforms exist; some are free while others require an initiation fee for the presenter. A synchronous online journal club could use any platform that allows online group chat, video and audio streaming, and screen sharing. Many institutions have paid subscriptions to one of these services. GoToMeeting was used for the first eight sessions based on availability of an institutional subscription. Zoom and Google Hangouts have also been used with equal success. None of these platforms incur any cost for the attendee. Sessions can be easily recorded with these platforms. However, we decided not to record the journal club discussions, as we felt participants could have been more hesitant to speak and share their analysis and opinion if they were being recorded.

A few ground rules were reviewed prior to each session (Table). The rules exist to remind all participants that this journal club is for professional scholarly discussion. The rules help to clearly set the expectation that unprofessional criticism would not be tolerated. Professional behavior is particularly important in the online setting, where the conversation is broadcast to a larger group and the exact audience is not always known.

Due to the nature of the problem this educational innovation solves, the learner group may not be immediately available or easy to contact. Therefore, some advertising and recruitment of learners is necessary. A network of simulation fellows and scholarly professionals were invited to participate by using a multiplatform approach. Advertisement strategies included directly e-mailing fellowship program directors, posting to discussion boards, and announcing events on social media (Twitter, Instagram, and Facebook). Fellowship directors were targeted as the initial person of contact as they have more visible and stable contact information. Fellows and trainees frequently have changing contact information due to institutional changes during their early career. Senior faculty were also invited to serve as faculty mentors.

In an effort to enhance the educational value of this initiative, the participants were given a program evaluation survey after each session. Additional data collected included demographics and educational opportunities available locally. Participants were also asked to provide feedback to the presenter. Initial data was collected using an online survey tool through the learning management system at the Winter Institute for Simulation, Education, and Research (WISER; Pittsburgh, PA) (Appendix).

## IMPACT/EFFECTIVENESS

The synchronous online journal club is an educational innovation connecting simulation fellows across geographic barriers. This electronic education activity allows scholarly exchange using existing technology that is cheap and readily available. The broad geographic and subspecialty participation demonstrates the easy accessibility of this format. The initial eight sessions were hosted by WISER. In this article, we focus on presenting data from these eight sessions, which ran from October 2014 to June 2016. Average attendance at these sessions was 10, with a range of 6–15.

**Figure 1:**
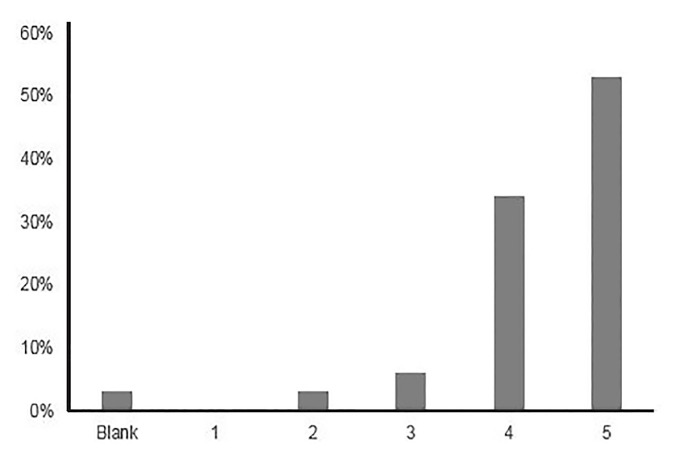
Participant responses on a Likert scale ranging from 1 (poor) to 5 (excellent) in response to the question “Please rate this overall program” (n=32).

**Figure 2:**
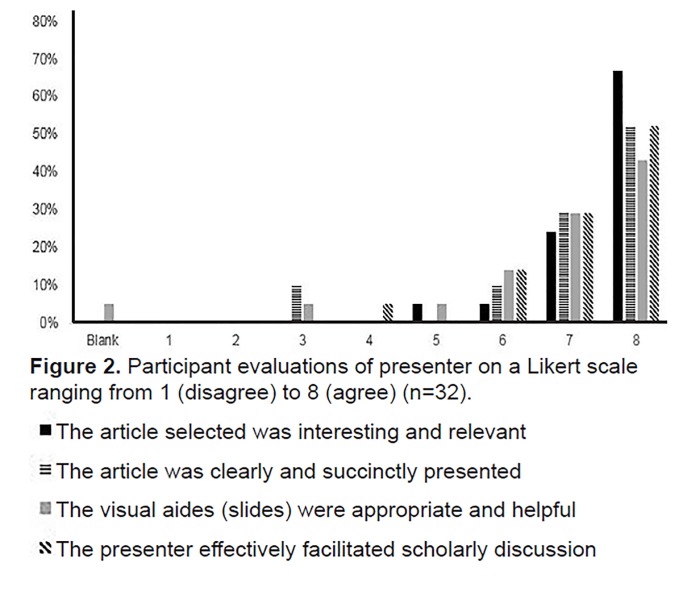
Participant evaluations of presenter on a Likert scale ranging from 1 (disagree) to 8 (agree) (n=32). ■ The article selected was interesting and relevant

Participants were from various clinical specialties including emergency medicine, anesthesia, surgery, pediatrics, biomedical engineering, critical care, internal medicine, nursing, and pharmacy. The majority of respondents identified as simulation fellows, with a minority identified as researchers, administrators, or simulation center directors. All participants had a particular interest in simulation. The geographic spread included participants from California, Washington State, Massachusetts, New York, Pennsylvania, Florida, Texas, Ohio, Michigan, Illinois, North Carolina, Washington DC, and Canada. Participants were asked to rate the overall journal club (Figure 1) and provide feedback to the presenter. (Figure 2) Comments to the prompt “Please provide any general comments” included the following: “Great discussion of relevant articles”; “Well-organized, informative, and thoughtful journal club”; and “Great idea to connect sim folks from across the country!”

This innovation broadened the exposure of fellows to journal club, as many participants did not have this available locally. Participation in journal club has been shown to increase scholarly reading behavior.^4^ Satisfaction scores indicated a positive response with few technology issues. This program provided a cost-effective way to encourage scholarly activity and collaboration. The feedback indicated a level of quality in the presentation. A key role in developing high-quality content is appropriate mentorship to aid with article selection and presentation preparation. Another important element is providing resources such as the slide template to guide the presentation format.

While this program provides great value, there are some challenges to creating a synchronous online journal club. As a live video conference, audience participation is somewhat limited by times zones. For example, 8 am might work great for those in Eastern Standard Time (EST), but this translates to 5 am in Pacific Standard Time. When scheduling journal club events, the leader of the program needs to choose a time that is reasonable to the greatest audience. Through experimentation and informal feedback, we found that 2 pm EST worked the best for participants across U.S. time zones. It is late enough in the day for those on the West Coast to attend after working a late evening shift the night before, but still during the “normal office hours” for those on the East Coast.

The distribution of articles can pose a challenge. Due to copyright concerns, we only distributed citations and links to PubMed. Most participants are able to access the articles for no cost, as they belong to academic institutions with subscriptions to most journals. However, this must be recognized as a potential limitation. Another potential concern is the recruitment of presenters. While any academically minded simulation educators or researchers are welcome to attend the sessions and contribute to the conversation, fellows are identified as presenters. There is generally a steady stream of fellows willing to present. This pool of fellows is refreshed every year or two, as they graduate and are replaced by new trainees. For the fellows, presenting during journal club is an opportunity to increase their visibility in front of a diverse audience while providing a platform for networking and allowing for honing of presentation skills. The journal club also provides publicity for their training program.

## LIMITATIONS

Several limitations of this study need to be addressed. In the first eight sessions, we had a total of 83 learner encounters. We collected 32 survey responses, for a response rate of 39%. Feedback forms were completely anonymous; thus, because attendance records did not allow for us to track the percentage of repeat attendees, they could not be excluded from the data. The feedback form was locally developed, and no formal validation occurred. The qualitative comments shared in this article are descriptive and representative of the feedback received; however, no formal thematic analysis was performed and was beyond the scope of this project.

## CURRENT STATE/FUTURE DIRECTION

Now that the program has been established, it began to be supported by the Society for Simulation in Healthcare (SSH) as an affinity group in 2018. This allows for easier communication to schedule and promote upcoming events through a common website and discussion board. Anyone can create a free account, as it does not require membership in SSH. Current feedback is collected using Google Forms. Dates are now announced annually, at the beginning of the academic year to allow ample time for schedule requests.

In conclusion, the educational experience of subspecialty trainees can be enhanced by using low-cost, existing technology to connect peer learners and passionate experts with an online journal club. Niche training programs, such as simulation fellowships, are not ubiquitous and therefore geographically dispersed. This synchronous journal club was trialed using simulation fellows, and would likely have success in other subspecialties that face similar challenges.

## Supplementary Information



## Figures and Tables

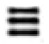
 The article was clearly and succinctly presented

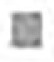
 The visual aides (slides) were appropriate and helpful

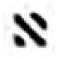
 The presenter effectively facilitated scholarly discussion

**Table:** Ground rules shared at the beginning of each online journal club.

Ground rules
Critical review of research in this forum is meant for educational purposes and to promote analytic thought. It by no means is meant to offend or devalue the research presented, the authors, or participants.
Please keep your comments constructive and professional.
Please use headphones and mute your microphone when not speaking to reduce feedback.
