# Supplementary material for: Synchronous Online Journal Club to Connect Subspecialty Trainees across Geographic Barriers
Source: West J Emerg Med. 2020 Dec 9;21(1):33–6. doi: 10.5811/westjem.2019.7.43545 (PMC6948697; doi:10.5811/westjem.2019.7.43545)
Supplement: Supplementary file 1 [file wjem-21-33-s001.pdf]

Sim Fellow Journal Club- Feedback Form

Please rate this overall program:

POOR EXCELLENT

○ ○ ○ ○ ○

Please provide any general comments:

Does the center that you work at have a journal club?

a. Yes, with a focus on simulation
b. Yes, with another focus
c. No

If your center has a journal club with another focus, please specify:

What time is most convenient for you to attend this meeting?

	Eastern	Central	Mountain	Pacific
A	10:00am	9:00am	8:00am	7:00am
B	12:00pm	11:00am	10:00am	9:00am
C	2:00pm	1:00pm	12:00pm	11:00am
D	4:00pm	3:00pm	2:00pm	1:00pm
E	6:00pm	5:00pm	4:00pm	3:00pm

What day is most convenient for your attend this online meeting?

a. Monday
b. Tuesday
c. Wednesday
d. Thursday
e. Friday
f. Saturday
g. Sunday

On average, how many simulation related journal articles do you read every month?

On average, how many times do you communicate with a peer at a different simulation center every month?

Thank you for taking the time to provide this valued feedback. Please provide the following information so we can better tailor this journal club to your interests.

I am particularly interested in Simulation as it relates to:

a. Education
b. Assessment
c. Research
d. Other

If other, please specify:

Which of the following best describes you:

a. Simulation Fellow
b. Resident
c. Simulation Center Director
d. Researcher
e. Administrator
f. Other

If other, please specify:

My credentials include:

a. MD
b. DO
c. PhD
d. CNS
e. RN
f. NP
g. PA
h. Other

If other, please specify:

My clinical specialty is best described as:

a. Critical Care
b. Emergency Medicine
c. EM/IM
d. Internal Medicine
e. Family Medicine
f. OB/GYN
g. Surgery
h. Psychiatry
i. Other

If other, please specify:

How many years post residency training are you:

a. Not residency trained
b. Currently in residency training
c. 1
d. 2
e. 3
f. 4
g. 5
h. More than 5

Did you experience any technical difficulties with the online meeting format or accessing the online materials?

Yes or No

Provide Feedback to Today's Presenter by Answering the Following the Questions:

The article selected was interesting and relevant

DISAGREE AGREE

The article was clearly and succinctly presented

DISAGREE AGREE

The visual aides (slides) were appropriate and helpful

DISAGREE AGREE

The presenter effectively facilitated scholarly discussion

DISAGREE AGREE

Please submit any constructive comments that will help the presenter continue to improve his or her skills presenting and facilitating small groups

Comments for the presenter:
